# The *Rosmarinus* Bioactive Compound Carnosic Acid Is a Novel PPAR Antagonist That Inhibits the Browning of White Adipocytes

**DOI:** 10.3390/cells9112433

**Published:** 2020-11-07

**Authors:** Cécilia Colson, Pierre-Louis Batrow, Nadine Gautier, Nathalie Rochet, Gérard Ailhaud, Franck Peiretti, Ez-Zoubir Amri

**Affiliations:** 1 Université Côte d’Azur, CNRS, Inserm, iBV, 06103 Nice, France; Cecilia.COLSON@univ-cotedazur.fr (C.C.); Pierre-Louis.Batrow@unice.fr (P.-L.B.); Nadine.Gautier@univ-cotedazur.fr (N.G.); rochet@unice.fr (N.R.); ailhaud@unice.fr (G.A.); 2 Aix Marseille Université, INSERM, INRAE, C2VN, 13007 Marseille, France; Franck.PEIRETTI@univ-amu.fr

**Keywords:** carnosic acid, PPA, adipocyte, conversion, antagonist, browning

## Abstract

Thermogenic brown and brite adipocytes convert chemical energy from nutrients into heat. Therapeutics that regulate brown adipocyte recruitment and activity represent interesting strategies to control fat mass such as in obesity or cachexia. The peroxisome proliferator-activated receptor (PPAR) family plays key roles in the maintenance of adipose tissue and in the regulation of thermogenic activity. Activation of these receptors induce browning of white adipocyte. The purpose of this work was to characterize the role of carnosic acid (CA), a compound used in traditional medicine, in the control of brown/brite adipocyte formation and function. We used human multipotent adipose-derived stem (hMADS) cells differentiated into white or brite adipocytes. The expression of key marker genes was determined using RT-qPCR and western blotting. We show here that CA inhibits the browning of white adipocytes and favors decreased gene expression of thermogenic markers. CA treatment does not affect β-adrenergic response. Importantly, the effects of CA are fully reversible. We used transactivation assays to show that CA has a PPARα/γ antagonistic action. Our data pinpoint CA as a drug able to control PPAR activity through an antagonistic effect. These observations shed some light on the development of natural PPAR antagonists and their potential effects on thermogenic response.

## 1. Introduction

Mammalian adipose organs can be divided into two distinct types of adipose tissues: white and brown. White adipose tissue (WAT) is specialized in the storage and release of fatty acids [1], which are required as an energy source for heart and muscles. In contrast, brown adipose tissue (BAT) dissipates energy in the form of heat by uncoupling the mitochondrial respiratory chain from ATP synthesis [2,3]. Adipose tissue is the largest endocrine organ and links metabolism and immunity [4]. It is a major actor in the regulation of energetic metabolism and represents a potential therapeutic target to combat fat mass disorders such as obesity and hypermetabolism in critical illness.

Obesity has become a worldwide socioeconomic burden and constitutes a substantial risk factor for hypertension, type 2 diabetes, cardiovascular diseases, and certain cancers [5,6]. Despite enormous efforts, there is no efficient treatment for patients with obesity, except bariatric surgery for morbidly obese patients. Various lifestyle interventions and proposed pharmacological remedies have not yet been shown to induce sustained body weight reduction with long-term benefits to the health and well-being of patients with obesity [7].

Critical illnesses lead to more than 50% of deaths in the first week and may be due to sepsis, burns, or cancer-induced cachexia, which induce a negative energy balance with hyper-activation of BAT and high recruitment of new thermogenic adipocytes located within WAT, known as beige or brite adipocytes (brown-in-white) [8]. High increases in catecholamine production, such as epinephrine and norepinephrine, and pro-inflammatory factors are observed in critical illness, but pharmacologic treatments are associated with secondary effects such as gastrointestinal and cardiovascular failures. Obesity appears to be protective against death induced by critical illness, and this process is called the “obesity paradox”.

Adipocytes with thermogenic characteristics located within WAT are known as brite (brown-in-white) or beige adipocytes and are found as islets that can be induced in response to cold, β-adrenergic, or peroxisome proliferator-activated receptor (PPAR) agonists and to other stimuli [9,10,11]. The number of brown and brite adipocytes declines with age and with the development of obesity [12,13]. Identification of cellular and molecular mechanisms involved in the formation and activation of brite adipocytes may lead to the development of novel therapeutic tools. In this respect, several compounds, some of which are natural compounds, have already been reported to play key roles [11,14]. PPARs play critical roles in white and brown adipogenesis and function and, thus, represent key targets [15,16,17]. To identify potential compounds that are able to control brown fat formation and/or activation, we focused on the active principles that are found in plants used in traditional medicine of which the leaves are traditionally consumed as tea or for topical treatments [14,18]. We focused our attention on carnosic acid (CA), a major active component from rosemary (*Rosmarinus officinalis*), a plant well known for its medicinal properties [19,20]. CA exhibits multiple bioactive properties including antioxidant, anti-inflammatory, and anticancer activities [21,22]. It also affects adipose tissue development and hepatic steatosis and improves cholesterolemia and glycemia [21,23]. Rosemary extracts containing mainly CA are used in Europe and Asia as an antioxidant in food additives [24]. CA was found to inhibit adipogenesis in rodent cell models such as 3T3-L1 by inhibiting clonal mitotic expansion and gene expression of master genes [25,26]. CA also impacts adipocyte differentiation and glucose transport [26,27]; however, the effects of CA on the formation and function of brown/brite adipocyte are presently unknown.

Herein, we report the role of CA in the browning of white adipocytes and in the inhibition of the expression of thermogenic markers of brite adipocytes in human and murine cell models.

## 2. Materials and Methods

### 2.1. Reagents

Cell culture media, insulin, and trypsin buffers were purchased from Invitrogen (Cergy Pontoise, France). Fetal bovine serum was from Eurobio (Les Ulis, France). hFGF2 was from Peprotech (Neuilly sur Seine, France), and other reagents were from Sigma-Aldrich Chimie (Saint-Quentin Fallavier, France) or Cayman (Montigny-le-Bretonneux, France).

### 2.2. Cell Culture and Stromal Vascular Fraction Preparation

#### 2.2.1. hMADS Cells Culture

The establishment and characterization of human multipotent adipose-derived stem (hMADS) cells have previously been described [28,29]. Briefly, these cells, which were isolated from white adipose tissue removed from surgical scraps of infants undergoing surgery, did not enter senescence while exhibiting a diploid karyotype, were non-transformed though they expressed significant telomerase activity, did not show any chromosomal abnormalities after 140 population doublings (PDs), and maintained their differentiation properties after 160–200 PDs. hMADS cells were able to withstand freeze/thaw procedures, and their differentiation could be directed under different culture conditions into various lineages [29]. The cell populations that have been studied were isolated from the pubic region fat pad of a 4-month-old male donor (hMADS3). Cells were seeded at a density of 5000 cells/cm^2^ in Dulbecco’s Modified Eagle’s Medium (DMEM) supplemented with 10% Fetal Bovine Serum (FBS), 15 mM Hepes, 2.5 ng/mL hFGF2, 60 mg/mL penicillin, and 50 mg/mL streptomycin. hFGF2 was removed when cells reached confluence. Differentiation was induced in cells in a serum-free medium at day 2 post-confluence (designated as day 0) in DMEM/Ham’s F12 (1:1) media supplemented with 10 µg/mL transferrin, 10 nM insulin, 0.2 nM triiodothyronine, 1 µM dexamethasone, and 500 µM isobutyl-methylxanthine for 4 days. Cells were treated between days 2 and 9 with 100 nM rosiglitazone (a PPARγ agonist) to enable white adipocyte differentiation. At day 14, conversion of white to brite adipocytes was induced by a second rosiglitazone treatment for 4 days. Alternatively, for some experiments, cells were treated with GW7647, a PPARα agonist. Media were changed every other day, and cells were used at the indicated days.

#### 2.2.2. Mouse Adipose Tissue SVF Preparation and Culture

Twelve-week-old C57Bl/6J mice were euthanized by cervical dislocation to isolate stromal vascular fractions (SVF), as described previously [30,31,32]. Briefly, interscapular BAT depots were sampled, washed in PBS, and minced. Adipose tissue samples were digested for 45 min at 37 °C in DMEM containing 2 mg/mL collagenase A (Roche Diagnostics, Meylan, France) and 20 mg/mL bovine serum albumin (Sigma-Aldrich Chimie, Saint-Quentin Fallavier, France). Lysate was successively filtered through 250-, 100-, and 27-µm nylon sheets and centrifuged for 5 min at 500× *g*. The pellet containing stromal vascular fraction (SVF) cells was subjected to a red blood cell lysis procedure. SVF cells were plated and maintained in DMEM containing 10% FBS until confluence. Differentiation was induced in the same medium supplemented with 1 µM dexamethasone, 0.5 mM isobutylmethylxanthine, and 170 nM insulin for two days. Cells were then maintained for 7–10 days in DMEM containing 10% FCS in the presence of 10 nM insulin and 2 nM triiodothyronine for white and 1 µM Rosiglitazone for brown adipogenesis. Media were changed every other day.

### 2.3. Isolation and Analysis of RNA

These procedures followed Minimum Information for Publication of Quantitative Real-Time PCR Experiments (MIQE) standard recommendations and were conducted as described previously [33]. The oligonucleotide sequences, designed using Primer Express software, are shown in Supplementary Table S1. Quantitative PCR (qPCR) were performed using SYBR qPCR premix Ex TaqII from Takara (Ozyme, Montigny-le-Bretonneux, France), and assays were run on a StepOne Plus ABI real-time PCR machine (PerkinElmer Life and Analytical Sciences, Boston, USA). The expression of selected genes was normalized to that of the 36B4 housekeeping gene and then quantified using the comparative-ΔCt method.

### 2.4. Lipolysis Assays

Lipolysis was assessed by measuring glycerol release [34] from differentiated cells as previously described [28,35]. Differentiated adipocytes were insulin-deprived for 30 min; then, fresh medium was added. The cells were immediately subjected to 1 µM isoproterenol, a pan β-adrenergic receptor agonist for 90 min. The sampled medium was used to measure glycerol release with a free glycerol reagent (Sigma-Aldrich Chimie, Saint-Quentin Fallavier, France), according to the manufacturer’s instructions. The results were normalized to the protein amount.

### 2.5. Western Blot Analysis

Proteins were extracted from cells or tissues as previously described [36]. Equal amounts of cellular proteins, 30 to 50 µg, were separated by electrophoresis using gradient gels (4–15%) and blotted onto PVDF membranes. Following blocking, membranes were incubated. Primary antibody incubation was performed overnight at 4 °C (anti-UCP1, Abcam #ab10983, dilution 1:1000; anti PPARγ, CST #2430, dilution 1:1000; and anti-TBP, CST #D5C9H, dilution 1:1000). Primary antibodies were detected with HRP-conjugated anti-rabbit or anti-mouse immunoglobulins (Promega, Charbonnieres Les Bains, France). Detection was performed using Immobilon Western Chemiluminescent HRP Substrate (Merck-Millipore, Molsheim, France). Chemiluminescence obtained after adding Pierce ECL western blotting substrate (Thermo Scientific, Asnièrse sur Seine) was detected using an Amersham Imager 600 and quantified with Image Lab 5.0 software (Bio-Rad, Marnes-la-Coquette, France).

### 2.6. Cell-Based PPAR Transactivation Assay

HEK293 cells were transfected with Peroxisome Proliferator Response Element (PPRE)-driven Firefly luciferase (addgene 1015) and SV40-driven Renilla luciferase coding vectors together with PPARγ or PPARα expression vector. PPARγ-LBD-Gal4 or PPARα-LBD-Gal4 expression vector (given by Dr. Teruo Kawada, Kyoto University, Japan) was transfected along with the SV40-driven Renilla luciferase expression vector in HEK293 cells stably expressing the Gal4 response element-driven firefly luciferase reporter (pGL4.35(luc2P/9XGAL4UAS/Hygro) vector from Promega, Madison, WI, USA). Thirty-six hours after transfection, cells were exposed to the tested compounds for an additional 16 h; then, firefly and Renilla luciferase activities were measured in the cell lysates using the reagents Genofax A and C (Yelen) in an EnSight multimode reader (Perkin Elmer, Courtaboeuf, France). PPAR transactivation activity of the compounds is calculated as ratio of firefly to Renilla luciferase activity.

### 2.7. Statistical Analyses

Data are expressed as mean values ± SEM and were analyzed using InStat software (GraphPad Prism version 8.3.0 for Windows, GraphPad Software, San Diego, CA, USA). Data were analyzed by Student’s *t*-test or one-way or two-way ANOVA followed by a post hoc multi-comparison test (Tukey or Bonferroni). Differences were considered statistically significant when *p* < 0.05 in Student’s *t*-test. When ANOVA was performed, adjusted *p*-values < 0.05 were considered to assess statistical differences.

## 3. Results

### 3.1. Carnosic Acid Inhibits the Browning Process of White Adipocytes

In the first series of experiments, we investigated the effect of CA on adipogenesis using a well-characterized human cell model (hMADS: human Multipotent Adipose-Derived Stem) [28]. Treatment of hMADS cells with CA at the beginning of the differentiation process led to complete loss of adipogenesis (data not shown), in agreement with previous reported studies using mouse cell models [25,26].

**Figure 1:**
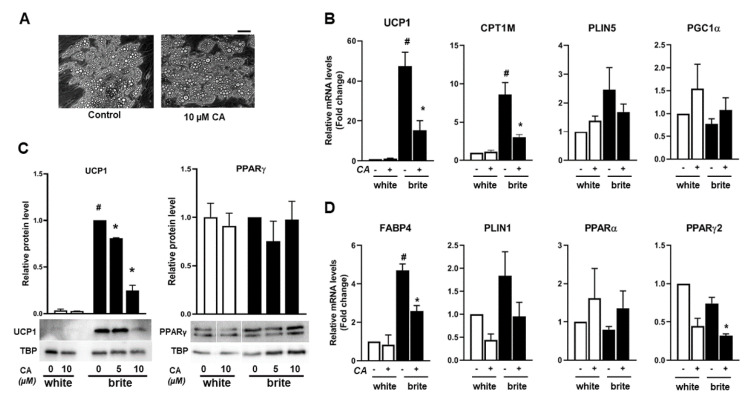
Carnosic acid (CA) inhibits the browning process of human white adipocytes. White human multipotent adipose-derived stem (hMADS) adipocytes were converted (from day 14 to day 18) into brite adipocytes using the peroxisome proliferator-activated receptor γ (PPARγ) agonist rosiglitazone in the absence (−) or the presence (+) of 10 µM CA (CA): (**A**) photomicrographs of differentiated hMADS cells treated or not with 10 µM CA, bar: 20 µm; mRNA levels of thermogenic (**B**) and adipogenic (**D**) markers; and (**C**) western-blot analysis using 40 (left panel) or 50 (right panel) µg total protein extracts. Histograms display mean ± SEM of three independent experiments; paired student, *p* < 0.05 was considered significant: #, white vs. brite adipocyte; *, untreated vs. CA treated condition.

In a second series of experiments, we tested if CA affected the formation and function of brite adipocytes. For this purpose, once differentiated into white adipocytes, hMADS cells, upon stimulation with PPAR agonists, can undergo the britening/browning process and can convert into functional brite thermogenic adipocytes [33,37]. Differentiation was induced in hMADS cells into white adipocytes for 14 days, and then conversion into brite adipocytes was induced for 4 days using rosiglitazone, a potent PPARγ agonist, in the absence or the presence of CA. CA is widely used in vitro from 1 to 10 µM [38], and we observed an optimal inhibitory effect on gene expression at 10 µM in our cell model using a range of 0–20 µM (Supplementary Figures S1 and S3). Up to 10 µM CA treatment, no effect on cell morphology and lipid accumulation was observed, discarding any cytotoxic effect (Figure 1A), whereas the 20 µM CA treatment was associated with cytotoxicity (data not shown). Gene expression analysis showed a pronounced decrease of mRNA levels of key thermogenic markers such as UCP1 and CPT1M in brite hMADS adipocytes treated with CA, whereas perilipin 5 (PLIN5) and PGC1α were only slightly affected (Figure 1B). UCP1 protein levels decreased upon CA treatment, which is consistent with gene expression data (Figure 1C) in brite hMADS adipocytes with an optimal inhibitory effect at 10 µM of CA.

The mRNA levels of adipocyte marker FABP4 were also significantly reduced (Figure 1D). We observed that the inhibitory effect of CA was more potent on brite adipocytes than on white adipocytes, indicating that PPARγ may play a key role. In line with this assumption, PPARγ2 mRNA levels were significantly reduced in the presence of CA whereas PPARα mRNA levels increased slightly but not significantly (Figure 1D). However, under these conditions, PPARγ protein levels were not affected (Figure 1C).

We had shown previously that activation of PPARα induces browning of white adipocytes [16]. In a similar manner to rosiglitazone-induced browning, GW7647 (a PPARα agonist) also induces gene expression of key thermogenic markers such as UCP1 and CPT1M and an adipogenic marker, FABP4, which was inhibited in the presence of CA (Supplementary Figure S1C,F).

CA’s effects on thermogenesis are not unique to human cells as we observed a similar inhibition in mouse primary adipocytes. Differentiation into adipocytes in stroma-vascular cells from brown adipose tissue of mice was induced in the absence or presence of rosiglitazone for 7 days, and cells were treated for the last 4 days with 10 µM CA. CA treatment did not modify UCP1 expression when cells were differentiated in the absence of rosiglitazone (Supplementary Figure S2). However, as expected, when UCP1 mRNA levels were induced in the presence of rosiglitazone, this induction was inhibited under 10 µM CA treatment (Supplementary Figure S2). Similar observations were obtained when using stroma-vascular cells derived from subcutaneous adipose tissue.

In conclusion, these results show that CA inhibits the expression of the UCP1 gene in human and mouse brown adipocytes.

### 3.2. Carnosic Acid Inhibits Thermogenic Marker Gene Expression of Human Brite Adipocytes

**Figure 2:**
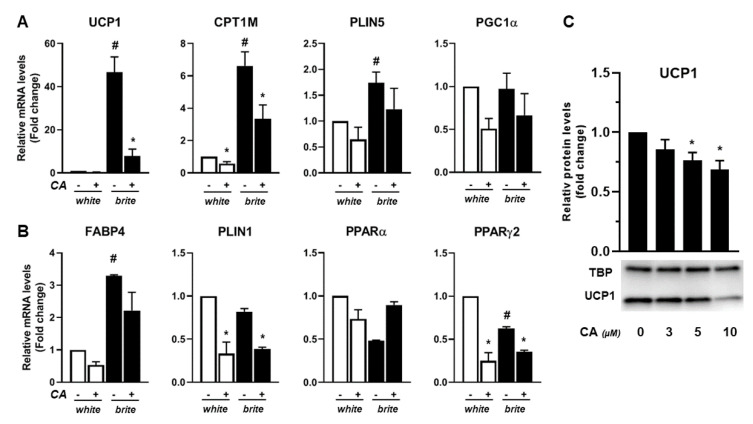
CA inhibits thermogenic marker gene expression of human brite adipocytes. White or brite hMADS adipocytes (at day 18) were maintained in the absence (−) or the presence (+) of 10 µM CA between days 18 to 22, and mRNA levels of thermogenic (**A**) and adipogenic (**B**) markers were analyzed. Forty micrograms of total protein extracts were analyzed by Western blot (**C**) representative of three experiments. Histograms display mean ± SEM of three independent experiments; paired student, *p* < 0.05 was considered significant: #, white vs. brite adipocyte; *, untreated vs. CA treated condition.

To investigate whether CA modulates the browning process, white and brite hMADS adipocytes (obtained following rosiglitazone treatment from days 14 to 18) were treated or not with 10 µM of CA at day 18 for 4 days. Cells were harvested and analyzed at day 22. CA treatment did not affect cell morphology and lipid accumulation, which rules out any cytotoxic effect (data not shown). The mRNA abundance of key thermogenic markers UCP1 and CPT1M (Figure 2A and Supplementary Figure S3B,E) and adipogenic markers FABP4 and PPARγ2 (Figure 2B) decreased in the presence of 10 µM CA, more in white adipocytes than in brite adipocytes. PLIN1 and PPARγ2 mRNA levels displayed a significant decrease (Figure 2B) in white and brite hMADS adipocytes upon CA treatment. PGC1α mRNA levels were not significantly affected by CA treatment (Figure 2A). Consistently, CA treatment induced a decrease of UCP1 at the protein level in brite hMADS adipocytes (Figure 2C).

In a similar way, CA inhibited the expression of the thermogenic marker of brown adipocytes obtained upon GW7647 treatment through downregulation of thermogenic and adipogenic markers, with an efficient dose at 10 µM CA (Supplementary Figure S3C,F).

Altogether, our results show that CA inhibits the browning process of white adipocytes by preventing (Figure 1) or inhibiting (Figure 2) the expression of key thermogenic markers.

### 3.3. Carnosic Acid Is a Potential Competitor of Rosiglitazone

**Figure 3:**
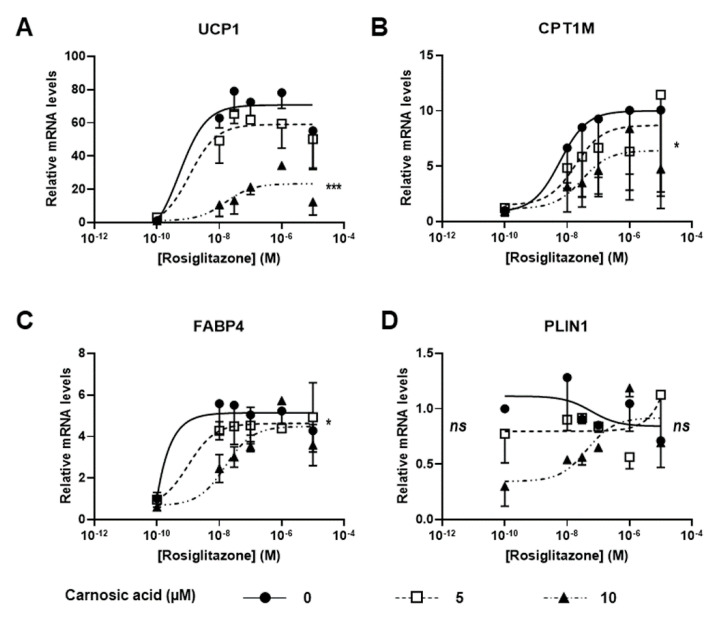
Efficiency of CA against rosiglitazone: conversion into brite adipocytes of white hMADS adipocytes was induced (between days 14 and 18) in the presence of varying amounts of rosiglitazone (10 nM–10 µM) in the absence (●) or the presence of 5 (□) or 10 (▲) µM CA. mRNA levels of thermogenic (UCP1 and CPT1M (**A**,**B**)) and adipogenic (FABP4, PLIN1, (**C**,**D**)) markers were analyzed. Each point on the curves represents mean ± SEM of two independent experiments (two replicates for each experiments); one-way ANOVA followed by Tukey post hoc test, the untreated vs. CA 10 µM-treated condition: *, adjusted *p* < 0.05; ***, adjusted *p* < 0.01.

Our aim was to test whether there was competition between rosiglitazone and CA. Thus, we evaluated the efficiency of the CA inhibitory effect. We treated white hMADS adipocytes during the browning process with various amounts of rosiglitazone in the absence or the presence of 5 and 10 µM of CA. White hMADS adipocytes were converted into brite adipocytes by rosiglitazone in a similar dose-dependent manner. UCP1 gene expression was maximal at 30 nM rosiglitazone, whereas a slight inhibition was observed at 10 µM rosiglitazone (Figure 3A). The effects observed are in agreement with affinity of rosiglitazone for PPARγ, with a Kd in the range of 10–50 nM [39]. In the presence of CA, the effect of rosiglitazone was slightly decreased at 5 µM CA and almost completely inhibited at 10 µM CA, i.e., more than 70% inhibition (Figure 3A). Similar observations, albeit to a lesser extent, were obtained with other thermogenic markers such as CPT1M (Figure 3B) and the adipogenic marker FABP4 (Figure 3C), whereas the expression of PLIN1, which is not a PPARγ target, was not affected (Figure 3D). Of note, in the absence of rosiglitazone, CA showed no effect on UCP1 expression (see Figure 1 and Figure 2 and data not shown).

Altogether, these observations show that CA inhibits the effects of rosiglitazone and thus might compete with rosiglitazone on PPARγ. Our data strongly suggest that CA behaves as an antagonist of rosiglitazone on PPARγ.

### 3.4. Carnosic Acid Antagonizes Rosiglitazone-Induced Activation of PPARγ

**Figure 4:**
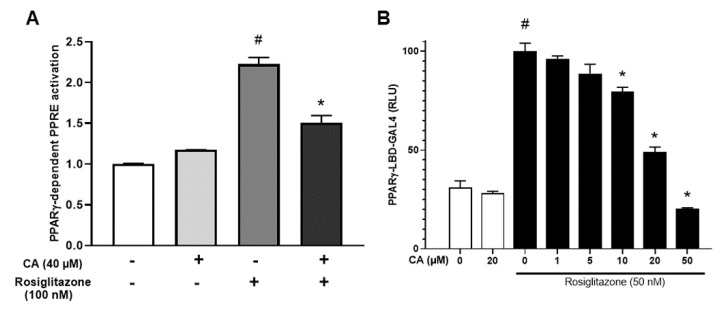
CA antagonizes rosiglitazone-induced activation of PPARγ. (**A**) HEK293 cells were transfected with PPRE-driven firefly luciferase and SV40-driven Renilla luciferase encoding vectors together with the PPARγ expression vector. Cells were then incubated in the presence of 100 nM rosiglitazone with or without 40 µM CA. PPRE promoter activity was calculated as the ratio of firefly/Renilla luciferase and expressed as fold over the control situation. (**B**) Concentration-dependent PPARγ transactivation activities of rosiglitazone were measured in the presence of varying amounts of CA using a PPARγ-LBD-GAL4 chimera assay. Values are expressed as % of the maximal response measured with rosiglitazone (50 nM). Data are displayed as mean ± SEM of two to three independent experiments (three replicates for each experiments). Student test, *p* < 0.05 was considered significant: #, rosiglitazone conditions vs. control; *, CA conditions vs. untreated.

To better understand the molecular mechanisms of CA inhibitory effects, we analyzed the effect on PPARγ transcriptional and transactivation activities in a cell system. For this purpose, in an initial series of experiments, a transcriptional activation assay (PPRE-based luciferase) was carried out to investigate the effect of CA on the direct transcriptional activity of full length PPARγ. PPARγ activation was measured by luciferase activity, reflecting the transcriptional activation of the PPRE driving luciferase in the presence of varying amounts of rosiglitazone and in the absence or the presence of 40 µM CA. As expected, rosiglitazone induced luciferase activity, and CA strongly inhibited the rosiglitazone-induced response (Figure 4A).

In the second series of experiments, we used a transactivation analysis based on the PPARγ-LBD-GAL4 chimera assay [40]. In this assay, the PPARγ ligand-binding domain was fused to the GAL4 DNA-binding domain and the firefly luciferase reporter gene was under the control of GAL4-binding elements (Figure 4B). In this system, CA did not affect the transactivation response of the luciferase reporter. Rosiglitazone treatment (50 nM) led to a strong transactivation of PPARγ, whereas CA treatment decreased the rosiglitazone-induced transaction in a dose-dependent manner (Figure 4B).

Our data show that CA targets the ligand-binding domain of PPARγ and affects the transcriptional activity by reducing the maximal response without affecting the half maximal effective concentration (EC50).

PPARα activation by GW7647 was also decreased in the presence of CA (Supplementary Figure S4). These results strongly suggest that CA inhibits PPAR ligand activation, potentially in a noncompetitive manner, and its effect seems to be stronger on PPARγ than on PPARα.

### 3.5. The Carnosic Acid Inhibitory Effect Is Normalized upon its Removal

**Figure 5:**
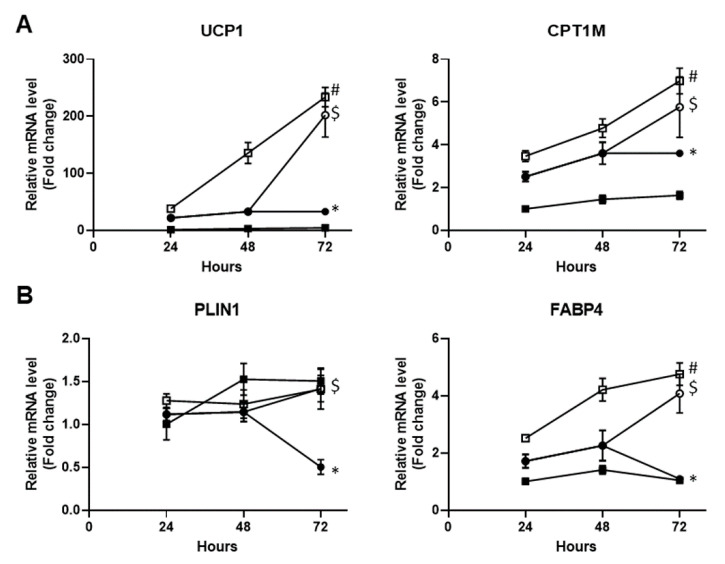
The CA inhibitory effect is normalized upon its removal. Conversion of white hMADS adipocytes (from day 14 to day 17) into brite adipocytes was induced with 100 nM rosiglitazone in the absence (□) or the presence (●) of 10 µM CA for 72 h. CA was removed for the last 24 h (◯). Untreated white hMADS adipocytes (■) served as the control. mRNA levels of thermogenic (UCP1 and CPT1M, **A**) and adipogenic (FABP4 and PLIN1, **B**) markers were analyzed. Data are displayed as mean ± SEM of two independent experiments (two replicates for each experiments). Two-way ANOVA followed by multi-comparisons and Bonferroni correction, adjusted *p* < 0.05 was considered significant: **#**, white vs. brite adipocyte; *, untreated vs. CA treated condition, $: significant for treated 72 h vs. 48 h.

We next checked whether the CA inhibitory effects were reversible. To answer this question, white hMADS adipocytes were treated with the PPARγ agonist rosiglitazone in the absence or the presence of CA for 72 h. CA was then removed or not for the last 24 h. As expected, rosiglitazone induced an increase in the expression of thermogenic markers UCP1 and CPT1M during the 3 days of treatment, that was inhibited in the presence of 10 µM CA (Figure 5A). Removal of CA for the last 24 h allowed full recovery of UCP1 and CPT1M mRNA levels (Figure 5A). Similar observations were obtained with the adipogenic markers FABP4 and PLIN1 (Figure 5B). Thus, it appears that the CA inhibitory effects on the browning process are reversible.

### 3.6. Effects of Carnosic Acid on Lipolysis

Lipolysis is one of the major adipocyte functions which enables the release of stored energy. Under β-adrenergic stimulation, the adipocyte hydrolyzes stored triglycerides into glycerol and free fatty acids. Importantly, given that thermogenic adipocytes use high amounts of fatty acids to produce heat, we tested whether CA affected lipolysis. For this purpose, brite hMADS adipocytes were treated or not with 10 µM CA for 4 days and stimulated by isoproterenol, a pan β-adrenergic receptor agonist in order to induce lipolysis.

**Figure 6:**
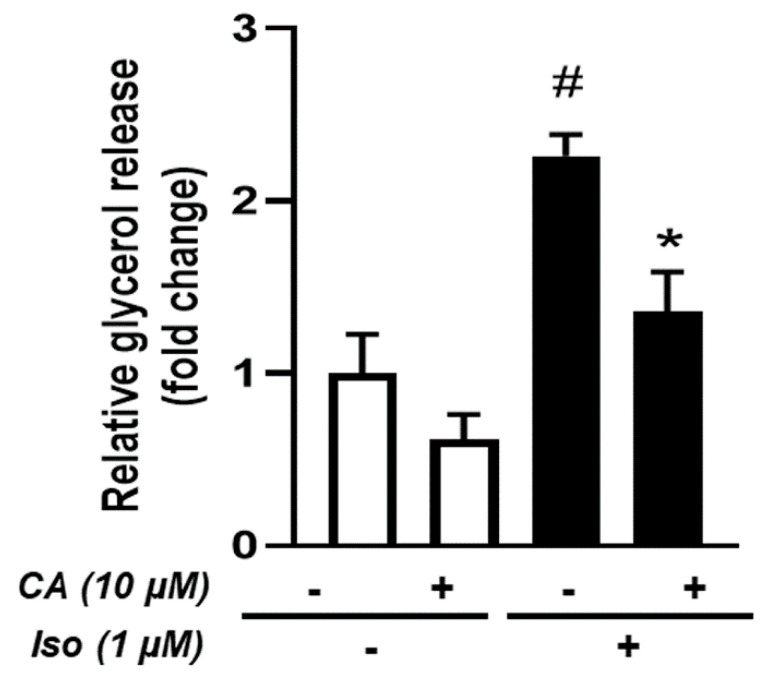
Effects of CA on lipolysis: conversion of hMADS adipocytes into brite adipocytes was induced in the absence (−) or the presence (+) of 10 µM CA for 4 days. Basal and isoproterenol-induced lipolysis were measured during the last hours. Data are displayed as mean ± SEM of three independent experiments; paired student, *p* < 0.05 was considered significant: #, no isoproterenol challenge (iso −) vs. isoproterenol treatment (iso +); *, untreated vs. CA-treated condition.

Our results showed a decrease in glycerol release when brite hMADS adipocytes were treated with CA (Figure 6). Nevertheless, the same reduction (around 2-fold) was observed under basal and isoproterenol-stimulated conditions, suggesting that CA decreased global lipolytic capabilities with no specific effect on β-adrenergic stimulation.

## 4. Discussion

The control of brown/brite adipocyte formation and function represents a promising therapeutic target to combat development and redistribution of fat mass and metabolic disorders. Several studies have shown that herbal medicine can play a role in the control of adipogenesis and metabolic diseases. Targets of herbal compounds that affect insulin signaling include the insulin receptor pathway and PPARs, among others [14,41,42]. Herein, we showed that CA, a major compound from rosemary, plays a PPARα/γ antagonist role inhibiting the browning process of white adipocytes and inhibiting thermogenic marker expression in brite adipocytes. PPARs represent a transcription factor family playing key roles in the regulation of important metabolic pathways and cellular functions in the pathophysiology of diabetes and obesity. So far, three different PPAR isoforms, designated α, β/δ, and γ, have been identified [43]. PPARγ is a master gene that drives adipogenesis, lipid metabolism, and browning process of white adipocytes [44,45]. The endogenous agonists of PPARs are a class of fatty acids and their derivatives that originate from the diet, de novo lipogenesis, and lipolysis [43]. Natural endogenous ligands have not yet been identified, but chemical ligands/activators such as thiazolidinediones (for example, rosiglitazone) are potent activators of PPARγ and have been used in a clinical setting for the treatment of insulin resistance [46]. However, these benefits are associated with secondary effects, such as fluid retention, body weight gain, bone loss, myocardial ischemia, diarrhea, and heart failure [46]. We had previously reported that human mature white adipocytes can acquire the brown fat cell properties upon PPARγ or PPARα activation [16,32]. The brown identity of a fat cell relies on its capacity to sustain thermogenesis, for example, possessing sufficiently high mitochondrial oxidative capacities associated with activation of the bona fide uncoupling protein, UCP1. Indeed, CA treatment of white hMADS adipocytes prevents the rosiglitazone-induced browning as measured by UCP1 mRNA and protein expression as well as another thermogenic marker, CPT1M. Moreover, CA treatment of brite hMADS adipocytes led to a loss of the UCP1 expression that is characteristic of the whitening process. The effects of CA are reversible, demonstrating that the inhibitory effects of CA did not result in disappearance of the thermogenic expression profile. CA antagonizes the effects of rosiglitazone in a dose-dependent manner leading to the reduction of maximal response at 10 µM and small effects on the half maximal doses. Depending on the gene, the effects are either very pronounced (UCP1), moderate (FABP4), or finally weak effects on adipogenic markers that are not PPAR targets (PLIN1). Similar effects of CA were observed when using mouse primary cultures of adipocyte precursors with lower inhibition, which might be due to the presence of fetal calf serum and the existence of potential proteins trapping CA.

Using transactivation assays, our data show that CA antagonizes rosiglitazone at the PPARγ ligand-binding domain. Regarding PPARα, the effects are less efficient with 15% inhibition compared to 50% for PPARγ. As these experiments were carried out in the presence of fetal calf serum, one cannot rule out the presence of a potential PPARα agonist leading to a high background response. Indeed, in the presence of CA, the background response was lower and the maximal response to PPARα (GW7647) was slightly inhibited (Supplementary Figure S4). Altogether, our observations indicate that CA is a noncompetitive PPAR antagonist with a higher affinity for PPARγ than for PPARα.

Several studies have shown that CA exerts an inhibitory effect on adipocyte differentiation [25,26,47] and might be a potent therapeutic agent of overweight/obesity and associated diseases [23,27,48,49]. It has been shown that the inhibitory effects of CA on adipogenesis can be mediated by inhibition of PPARγ and C/EBPs, affecting also the LIP/LAP ratio [25], although the main function of CA appears to be related to antioxidative features as a quencher of reactive oxygen species [50]. Other functions have been reported to act through the EGFR/MAPK pathway, Akt pathway, or induction of glutathione S-transferase [51,52,53] as well as inhibition of lipoxygenases that affect potential PPAR activators/ligands [54]. To our knowledge, our data represent the first demonstration that CA plays an antagonistic role against ligand binding on PPARγ and, therefore, may represent a potential new class of natural compounds with known pharmacological properties in the treatment metabolic disorders. Altogether, CA seems to control both adipogenesis and thermogenesis, and for the latter, it might be through tight control of thermogenic markers including the most important, UCP1. Under conditions where browning of white adipocytes is exacerbated, such as in critical illness after a severe burn injury or cachexia [8,55,56,57], CA treatment may represent a potential therapeutic option. Animal models and patients with severe burns develop hypermetabolism with massive browning of white adipose tissue, hepatic steatosis, and cachexia, which are harmful and have limited therapeutic treatments. In a similar manner, cancer-associated cachexia, a wasting syndrome, is associated with increased browning of white adipose tissue, which leads to higher thermogenesis with increased lipid mobilization and energy expenditure, further worsening the clinical situation and risk of death. Inhibition of white adipose tissue browning in burn and cancer patients represents a promising approach that can be potentially achieved with CA treatment alone or in combination with other approaches. In line with this, it has been suggested that CA is efficient against obesity-associated cancers, in particular against colon cancer [22]. Furthermore, it is known that PPAR activation by thiazolidinediones is associated with bone fracture [58], and we could speculate that CA treatment has the potential to restore bone mass, given that betulinic acid, a PPARγ antagonist, is known to favor osteogenesis at the expense of adipogenesis [59].

CA is a phenolic diterpene, and given its lipidic nature, one can hypothesize that CA is able to interfere with PPAR ligands in the ligand-binding domain in a reversible manner to regulate receptor activity. In the absence of rosiglitazone, CA was able to inhibit adipogenic marker expression, suggesting that CA might antagonize endogenously synthesized ligands/activators. We cannot rule out the possibility that CA might exert some effects on the recruitment of cofactors and/or affect PPAR modifications such as phosphorylation. Further studies are necessary to shed some light on the mechanisms of interaction of CA and PPARs. The development of a new generation of natural PPAR antagonists represents a promising approach to address selectivity and therapeutic concerns.

## 5. Conclusions

In this work, we showed for the first time that CA had a PPARα/γ antagonistic action in a human cell model. CA inhibited the browning process of white adipocytes and decreased the expression of thermogenic key markers, suggesting that CA is able to induce whitening in thermogenic adipocytes. Genes that are under the control of PPAR, harboring a PPRE in their promoter, were affected upon activation of PPARα/γ. CA did not affect the β-adrenergic response; however, it inhibited the lipolytic ability associated with brite adipocytes. Importantly, the effects of CA were fully reversible. More detailed studies are necessary to decipher the mechanisms behind these effects. Our data open new horizons in the development of natural PPAR antagonists with potential effects on metabolic disorders.

## Supplementary Materials

The following are available online at https://www.mdpi.com/2073-4409/9/11/2433/s1, supplementary Figures S1–S4, supplementary Table S1. Figure S1: CA inhibits the browning process of human white adipocytes; Figure S2: CA inhibits the browning process of mice primary adipocytes; Figure S3: CA inhibits thermogenic marker gene expression of human brite adipocytes; Figure S4: CA antagonizes rosiglitazone-induced activation of PPARα; Table S1: Sequence of primers used for gene expression analysis.
