# Supplementary material for: The Rosmarinus Bioactive Compound Carnosic Acid Is a Novel PPAR Antagonist That Inhibits the Browning of White Adipocytes
Source: Cells. 2020 Nov 7;9(11):2433. doi: 10.3390/cells9112433 (PMC7695189; doi:10.3390/cells9112433)
Supplement: Supplementary file 1 [file cells-09-02433-s001.pdf]

Supplementary material

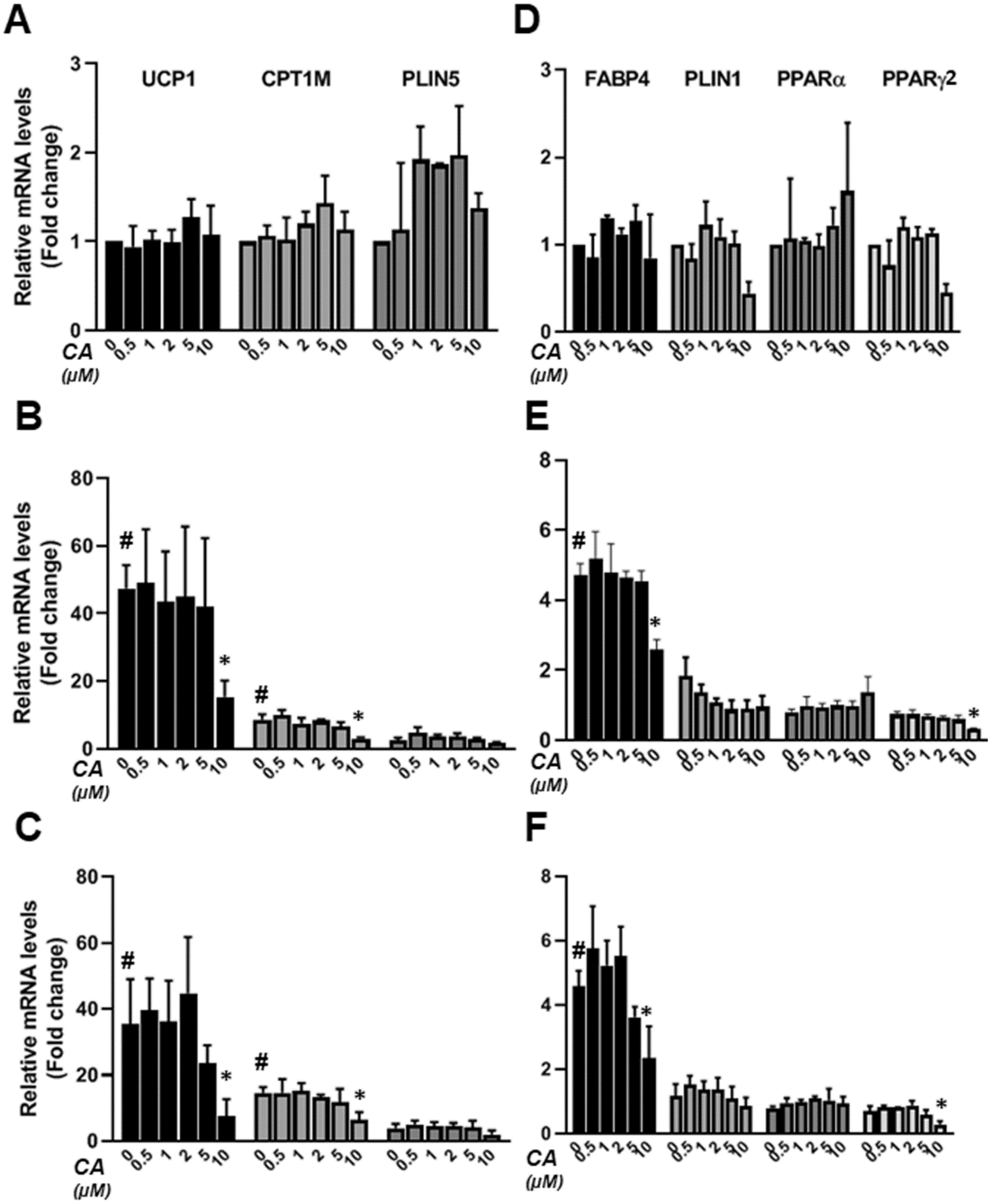

Supplementary Figure S1: CA inhibits the browning process of human white adipocytes. White hMADS adipocytes (A, D) were converted (days 14 to 18) into brite adipocytes with 100 nM rosiglitazone (B, E) or 300 nM GW7647 (C, F) in the presence of increasing CA concentrations. mRNA levels of thermogenic (A-C) and adipogenic (D-F) markers were analyzed. Paired student, $p<0.05$ considered as significant: #, white vs brite adipocyte; *, untreated *vs* CA treated condition.

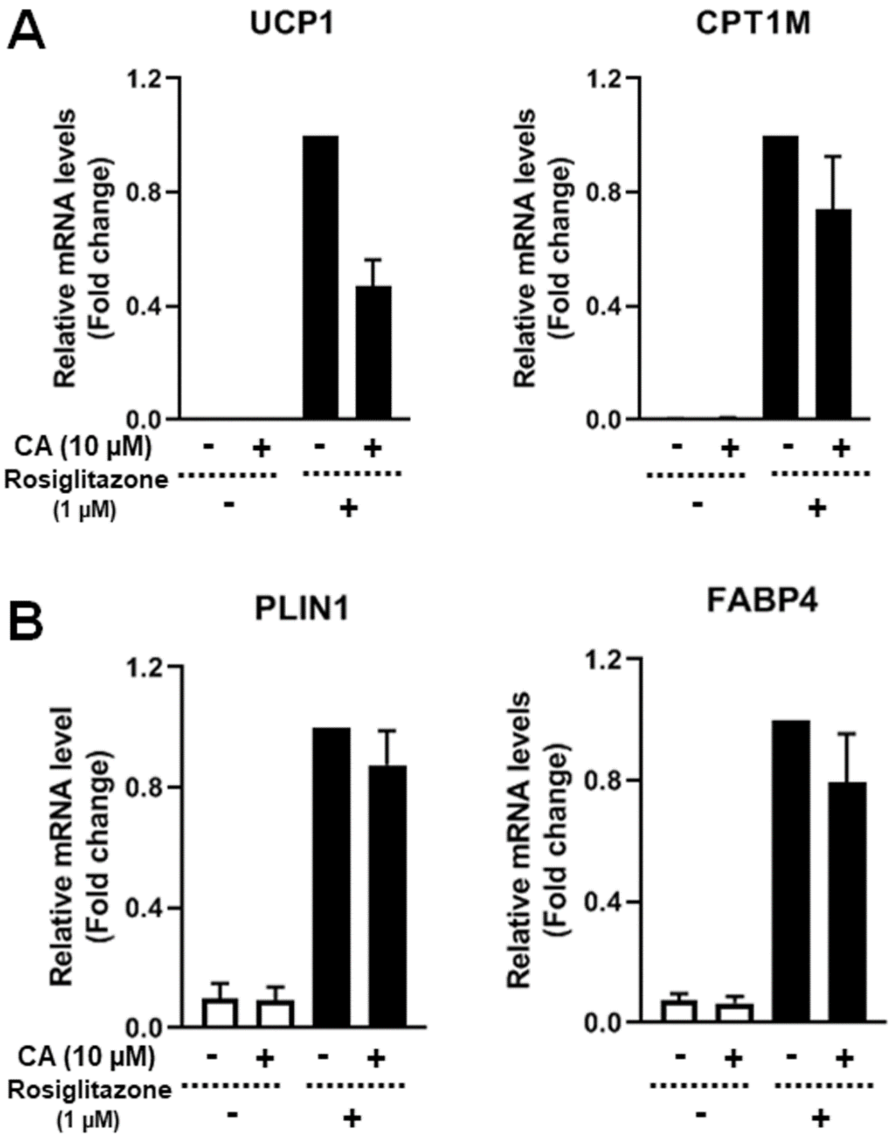

Supplementary Figure S2: CA inhibits the browning process of mice primary adipocytes. SVF cells derived from mice BAT were induced to differentiate into white or brown (rosiglitazone-treatment) adipocytes in the absence (-) or presence (+) of 10 µM of CA for the last 4 days and mRNA levels of thermogenic (A) and adipogenic (B) markers were analyzed. Histograms display mean ± SEM of three independent experiments.

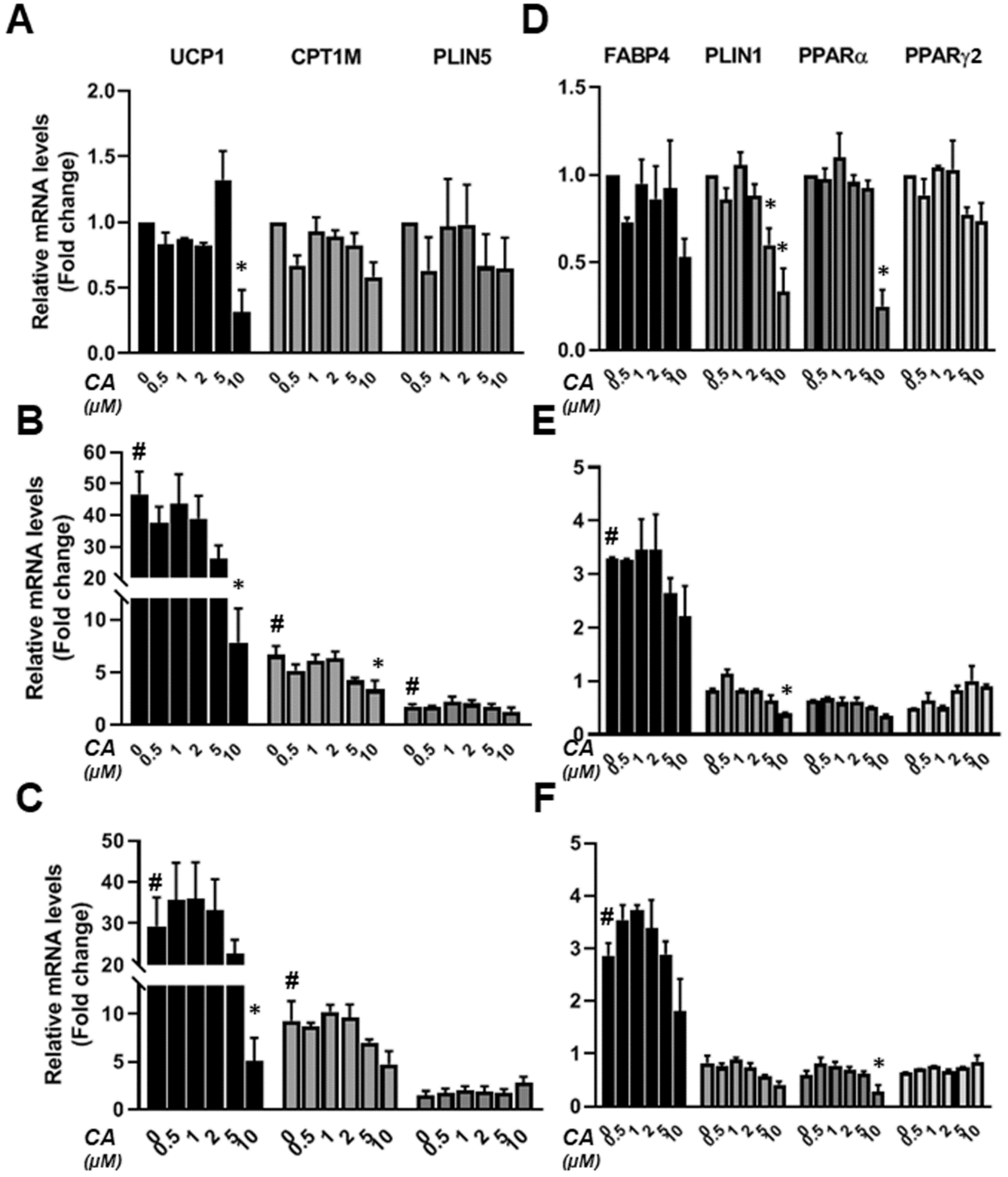

Supplementary Figure S3: CA inhibits thermogenic marker gene expression of human brite adipocytes. hMADS adipocytes were differentiated into white adipocytes (A, D), or brite adipocytes using rosiglitazone (B, E) or GW7647 (C, F) in the presence of increasing CA concentrations and mRNA levels of thermogenic (A-C) and adipogenic (D-F) markers were analyzed. Paired student, $p<0.05$ considered as significant: #, white *vs* brite adipocyte; *, untreated *vs* CA treated condition.

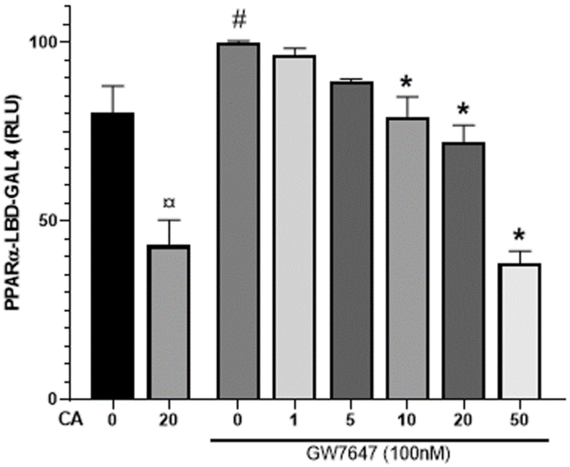

Supplementary Figure S4: CA antagonizes rosiglitazone-induced activation of PPARα. Concentration-dependent PPARα transactivation activities of GW7647 were measured in the presence of varying amounts of CA using a PPARα-LBD-GAL4 chimera assay. Values are expressed as % of the maximal response measured with GW7647 (100 nM). Data are displayed as mean ± SEM of two to three independent experiments (three replicates for each experiments). Paired student, $p<0.05$ considered as significant: #, GW7647 treated *vs* untreated; * and ¤, CA treated *vs* untreated.

Supplementary Table S1. Sequence of primers used for gene expression analysis.

Human oligonucleotide sequences		
**name**	**Reverse primer**	**Forward primer**
PPARγ2	ATCAGTGAAGGAATCGCTTTCTG	CAAACCCCTATTCCATGCTGTT
PPARα	TCCAAAACGAATCGCGTTGT	GGCGAACGATTCGACTCAAG
PGC1α	CTGTGTCACCACCCAAATCCTTAT	TGTGTCGAGAAAAGGACCTTGA
UCP1	CCAGGATCCAAGTCGCAAGA	GTGTGCCCAACTGTGCAATG
FABP4	CAACGTCCCTTGGCTTATGCT	TGTGCAGAAATGGGATGGAAA
CPT1-M	GAGCAGCACCCCAATCAC	AACTCCATAGCCATCATCTGCT
PLN1	GATGGGAACGCTGATGCTGTT	ACCCCCCTGAAAAGATTGCTT
PLN5	CTACGAGCACTCTGTGGGGA	GGTCTATCAGCTCCAGCGTCT
36B4	TGCATCAGTACCCCATTCTATCAT	AGGCAGATGGATCAGCCAAGA

Mice oligonucleotide sequences		
**name**	**Reverse primer**	**Forward primer**
36B4	TCC AGG CTT TGG GCA TCA	CTTTATCAGCTGCACATCACTCAGA
Cpt1m	GGCTCCAGGGTTCAGAAAGT	TGCCTTTACATCGTCTCCAA
Fabp4	CTTGTGGAAGTCACGCCTTT	AAGAGAAAACGAGATGGTGACAA
Ucp1	CACCTTCCCGCTGGACACT	CCTGGCCTTCACCTTGGAT
Pln1	AGCGTGGAGAGTAAGGATGTC	CTTCTGGAAGCACTCACAGG
